# Assessment of In Vitro Bioactivities of Polysaccharides Isolated from *Hericium* *Novae-Zealandiae*

**DOI:** 10.3390/antiox8070211

**Published:** 2019-07-08

**Authors:** Zhixia (Grace) Chen, Karen Suzanne Bishop, Hartono Tanambell, Peter Buchanan, Siew Young Quek

**Affiliations:** 1 Food Science, School of Chemical Sciences, The University of Auckland, Auckland 1010, New Zealand; 2 Auckland Cancer Society Research Centre, Faculty of Medical and Health Sciences, The University of Auckland, Auckland 1010, New Zealand; 3 Discipline of Nutrition and Dietetics, School of Medical Sciences, Faculty of Medical and Health Sciences, The University of Auckland, Auckland 1010, New Zealand; 4 Manaaki Whenua-Landcare Research, Auckland 1072, New Zealand; 5 Riddet Institute, New Zealand Centre of Research Excellence for Food Research, Palmerston North 4474, New Zealand

**Keywords:** polysaccharides, prostate cancer, anti-proliferation, AChE, antioxidant, *Hericiumnovae-zealandiae*

## Abstract

The objective of this study was to investigate the potential effect of the polysaccharides isolated from *Hericium novae-zealandiae*, a native New Zealand fungus, on the *in vitro* proliferation of prostate cancer cell lines, gene expression, acetylcholinesterase (AChE) activity, and oxidation. One water-soluble and two alkali-soluble polysaccharide fractions were isolated from *H. novae-zealandiae*. The proliferation of the prostate cancer cell lines DU145, LNCaP, and PC3 was evaluated following treatment with these polysaccharide fractions. It was found that the polysaccharides possess anti-proliferative activity on LNCaP and PC3 cells, with a 50% growth inhibition (IC_50_) value as low as 0.61 mg/mL in LNCaP. Subsequently, it was determined through via RT-qPCR assay that apoptosis was one of the possible mechanisms responsible for the anti-proliferative activity in LNCaP. This was supported by the up-regulation of *CASP3*, *CASP8*, and *CASP9*. An alternative, discovered in PC3, was revealed to be anti-inflammation, which was hinted at by the down-regulation of *IL6* and up-regulation of *IL24*. The polysaccharides also exhibited antioxidant and weak AChE inhibitory activities. This is the first report on the potential health benefits of polysaccharides prepared from the New Zealand fungus, *H. novae-zealandiae*.

## 1. Introduction

Mushrooms are known to possess bioactive compounds that are thought to enhance human health [1]. For centuries, mushrooms have been traditionally used as both a highly valued food and as a medicine [2]. In more recent years, the molecular mechanisms underlying the health benefits of mushrooms have gained interest. Several metabolites of mushrooms have been classified as active mycochemicals, namely proteins, polysaccharides, lipopolysaccharides, and glycoproteins [3]. Polysaccharides have been one of the most studied metabolites from mushrooms. They have been identified to possess substantial medicinal properties and have been implicated as a major active component of mushrooms [4]. 

*Hericium novae-zealandiae* (Colenso) Chr.A. Sm. and J.A. Cooper is a New Zealand native wood decay mushroom, traditionally consumed by Māori, who knew it as pekepekekiore [5]. Another species of *Hericium*, *H. erinaceus*, has been firmly established as an important medicinal mushroom as it exerts a wide range of activities, including anticancer [6,7,8,9], neuro-protection [10], antioxidant [11], and anti-inflammatory activities [12]. Polysaccharides isolated from *H. erinaceus* have been reported to be one of the major constituents responsible for anti-cancer, immuno-modulating, hypolipidemic, antioxidant, and neuro-protective activities of the species [13]. 

Very little is known about *H. novae-zealandiae* compared to its well-studied relative, *H. erinaceus*. Our previous published work has revealed that the New Zealand species is rich in nucleoside compounds [14]. In addition, we have recently isolated three liposoluble compounds, namely hericene B, ergosterol, and ergosterol peroxide, from the ethanol extract of *H. novae-zealandiae*. Furthermore, both the ethanol extract and ergosterol peroxide have been found to exhibit *in vitro* anti-proliferative activities on prostate cell lines in our recent work (manuscript submitted for publication). Information regarding the potential health benefits of the polysaccharides derived from *H. novae-zealandiae*, however, are lacking. The investigation of the potential bioactivities of its polysaccharides is, therefore, of interest, as polysaccharides have been considered important biomacromolecules, as demonstrated in *H. erinaceus* [15,16,17]. 

Polysaccharides isolated from *H. erinaceus* have been shown to possess anticancer activities in precancerous human gastric cells (MC) [18], human gastric cancer cell lines (SGC-7901) [19] and breast cancer cell lines (MCF-7), and HeLa cells [20]. For memory improvement related activity, neuroprotective effects against Aβ-induced neurotoxicity have been observed in rat pheochromocytoma PC12 cells after the treatment of two high molecular weight polysaccharides (1.7 × 105 Da and 1.1 × 105 Da) [21]. Therefore, since *H. novae-zealandiae* and *H. erinaceus* are congeneric, the same bioactivities might also be present in *H. novae-zealandiae*. Consequently, our hypothesis was that the polysaccharides isolated from *H. novae-zealandiae* might exhibit similar anti-cancer and memory improvement activities to that of *H. erinaceus*. Thus, determining suitable cancer cell lines and a memory improvement model for the polysaccharide fractions derived from *H. novae-zealandiae* is of great interest to test this hypothesis.

Prostate cancer (PCa) was selected as it is the most commonly diagnosed cancer in Australia and New Zealand and the fourth most common cancer worldwide [22]. According to a 2018 report from New Zealand’s Ministry of Health, 25.72% of all new cancer registrations and 12.66% of cancer deaths were attributed to PCa for New Zealand males [23]. Various phytochemicals, including polysaccharides, have been investigated for their capacity to combat cancer and to provide alternative cancer treatments to chemotherapeutic drugs [24].

LNCaP, DU145, and PC3 are the most frequently used cell lines in in vitro PCa studies [25]. The human embryonic kidney (HEK) 293 cell line was selected as the control non-cancer cell line. In the present study, the anti-proliferative activities of the polysaccharides of *H. novae-zealandiae* to three PCa and HEK293 cell lines were studied. In addition, the molecular mechanisms accounting for this activity were investigated through RT-qPCR assays.

With respect to the function of preventing memory impairment, a model from the cholinergic hypothesis was selected. This hypothesis claims that memory and learning impairment in Alzheimer’s disease (AD) patients are initiated by acetylcholine (ACh) deficiency [26]. Acetylcholinesterase (AChE) was found to be the enzyme responsible for regulating the level of synaptic ACh. Thus, the increase of acetylcholine levels in the brain resulting from the inhibition of AChE is one of the promising approaches for the treatment of AD [27]. This hypothesis has been supported by the fact that phytotherapy has been observed to alleviate AD symptoms by targeting the aforementioned cholinergic pathway [28]. 

In addition to the anticancer activity and improvement in cognitive impairment, the antioxidant activity of the polysaccharides was also measured in this study. Oxidation is a biological process resulting in free radicals, which attack living organisms during oxidative stress and trigger the development of aging and chronic degenerative diseases, including coronary heart disease, and cancers [27]. In addition, oxidative stress, resulting from the process of oxidation, has also been considered a major mechanism in the pathogenesis of dementia and other neurodegenerative diseases [29]. A therapy combining the inhibition of AChE and oxidation had been applied to modulate these kinds of cognitive decline [30]. Based on the fact that oxidation is related to the development of both cancer and memory impairment, the antioxidant capacity of polysaccharide fractions was measured to investigate the antioxidant potential of *H. novae-zealandiae*.

The present study aimed to screen the anticancer potential (and its molecular mechanisms) of, and the AChE inhibitory and antioxidant activities of, polysaccharides isolated from *H. novae-zealandiae*. 

## 2. Materials and Methods

### 2.1. Materials

All chemicals used in this study were of analytical grade, unless stated otherwise. The chemicals used in polysaccharide extraction and purification were absolute ethanol (ECP Ltd., Auckland, New Zealand), hydrochloric acid (Avantor, PA, USA), sodium hydroxide (Sigma-Aldrich, MO, USA), nitrogen (N_2_, BOC, Auckland, New Zealand), 1-butanol (Schartau Chemie, Barcelona, Spain, HPLC grade). The chemicals used in protein assays were bovine serum albumin (BSA, GIBCOBRL, CA, USA) and Coomassie Brilliant Blue G-250 (CBB, Bio-Rad Laboratories, CA, USA, Electrophoresis Purity Reagent). The chemicals used in the proliferation inhibition experiment were trichloroacetic acid (TCA), sulforhodamine B (SRB), glacial acetic acid (Emsure^®^) and disodium ethylenediaminetetraacetic acid (EDTA), all from Sigma-Aldrich, Auckland, New Zealand, as well as tris(hydroxymethyl)aminomethane (Tris, Invitrogen™), trypsin (Gibco™), and trypan blue dye (Gibco™), all from Thermo Fisher Scientific, Auckland, New Zealand. Phosphate buffered saline (PBS) was prepared in house. The chemicals used in the antioxidant experiment were 1,1-diphenyl-2-picrylhydrazyl (DPPH), 6-hydroxy-2,5,7,8-tetramethylchroman-2-carboxylic acid, 2,2’-azino-bis(3-ethylbenzothiazoline-6-sulphonic acid) (ABTS), potassium persulfate (K_2_S_2_O_8_), and 2,4,6-Tris(2-pyridyl)-s-triazine (TPTZ), all from Sigma-Aldrich, Auckland, New Zealand, as well as sodium acetate, acetic acid, ferric chloride (FeCl_3_), and sodium acetate (Na_2_CO_3_), all from ECP Ltd., Auckland, New Zealand. The chemicals used in the AChE experiment were AChE enzyme (*Electrophorus electricus* (electric eel), EC, Auckland, New Zealand), as well as trizma base, trizma HCl, eserine, DTNB (5,5’-Dithiobis-(2-Nitrobenzoic Acid), BSA, NaCl, MgCl_2_•6H_2_O, SDS (Sodium Dodecyl Sulphate), and acetylthiocholine iodide (ATCI), all from Sigma-Aldrich, Christchurch, New Zealand. Milli-Q water was prepared using a Milli-Q water purification system (Millipore Corporation, Burlington, USA). Freshly cultivated fruiting body specimens of *H. novae-zealandiae*, Auckland, May 2016 (ICMP 21483), were provided by a mushroom grower in Napier, New Zealand. The samples were frozen at −80 °C, freeze-dried, and reduced to a fine powder prior to analysis. 

### 2.2. Polysaccharide Extraction and Purification

Three polysaccharide fractions were obtained according to the method described by Jia’s group [31] with some minor modifications. Sixty grams of powdered sample was dispersed in 1 L of absolute ethanol overnight, followed by a reflux extraction at 60 °C to remove the lipid-soluble components. After centrifugation (5000 rpm/3864 *g*, 10 min, Sorvall Lynx 4000 Centrifuge, Thermo Fisher Scientific, New Zealand), the residue was subjected to extraction under agitation (500 rpm) with hot water (95 °C) for 3 h; the process was performed three times. The aqueous suspension was pooled and centrifuged (6000 rpm/5564 *g*, 10 min, Sorvall Lynx 4000 Centrifuge, Thermo Fisher Scientific, New Zealand) and the residue was reserved for the extraction of two other polysaccharide fractions. The supernatant was concentrated under vacuum at 60 °C and then a three times volume of absolute ethanol was added to induce precipitation at 4 °C overnight. The precipitated crude polysaccharides were collected after centrifugation performed as above. The precipitate was again dissolved in 100 mL water and dialyzed using dialysis tubing (Medical International Ltd., Mansourieh, Lebanon, molecular weight cut-off 12,000 Da) against water for 72 h to remove low molecular weight carbohydrate constituents. After concentration under vacuum at 60 °C, the contents of the dialysis tubes were treated with Sevag reagent (1-butanol:chloroform = 1:5) [32] at a ratio of 1:1 (volume to volume) three times to remove protein. After full oscillation and centrifugation, the supernatant solution was collected and combined for freeze drying at −80 °C in a vacuum freeze dryer to obtain the water-soluble polysaccharide (WCP) fraction.

The aforementioned residue was further extracted by suspending under agitation (500 rpm) with 1N NaOH in a nitrogen-filled container for 2 h and the process was then repeated twice. The supernatant was pooled and 6 N HCl was added to make the solution neutral. Then, the steps of concentration, dialysis, and centrifugation described above in obtaining WCP were repeated to obtain supernatant and precipitate. The supernatant was then treated with Sevag reagent and the precipitate with ethanol followed by freeze drying to obtain base-soluble polysaccharides (BP1). The precipitate was washed in water several times and then treated with Sevag reagent and lyophilized in a vacuum freeze dryer to obtain base-polysaccharides (BP2). Three polysaccharide fractions were dissolved in water or 0.2 N NaOH at different concentrations, depending on the requirements of each assay.

### 2.3. Protein Content in Polysaccharides

The content of protein in the polysaccharide fractions was determined by the method of Sevag [33] with minor modifications. The sample solutions were prepared by dissolving 10 mg of WCP in 10 mL water and by dissolving 10 mg of BP1 and BP2 into 0.2 N NaOH, respectively, using water or 0.2 N NaOH as the reagent blank accordingly. The protein content was calculated using a calibration curve. The analysis was performed in triplicate.

### 2.4. Anti-Proliferation Assay

Three human PCa cell lines PC3, DU145, and LNCaP, as well as a non-cancer HEK293 cell line, were provided by the Auckland Cancer Society Research Centre (ACSRC). These cell lines were authenticated by short tandem repeats by DNA Diagnostics prior to assay. These cell lines were cultured in Minimum Essential Medium supplemented with 5% fetal calf serum and 1% penicillin/streptomycin/glutamine in Becton Dickinson Falcon™ cell culture flasks (BD Biosciences) at 37 °C with a 5% carbon dioxide atmosphere.

All cell lines were seeded in experimental 96-well plates at a cell density of 2500 cells per well and incubated under the previously described conditions to ensure adherence to the bottom of the wells. The cells were then treated with the three polysaccharide fractions at gradient concentrations through a series of dilutions. WCP and BP1 were dissolved in water to prepare stock solutions of 40 and 30 mg/mL, respectively. The anti-proliferative assay of BP2 was not successful as BP2 is hardly soluble in water and both DMSO and 0.2 N NaOH, serving as the solvent to dissolve BP2, caused interference at the effective concentration of BP2. The dosing regimen of this experiment is shown in Table 1. Thereafter, the plates were placed in an incubator under the same conditions and given sufficient time for four doubling cycles. Cellular cytotoxicity was measured using the Sulforhodamine B-based assay [34]. The absorbance readings were measured at a wavelength of 490 and 450 nm with an EL808 Microplate Reader (Bio-Tek Instruments, Winooski, USA). Dose-response curves were generated using SPSS. The 50% growth inhibition (IC_50_) values of polysaccharides in each cell line were determined using dose-response curves. The experiment was performed using four technical repeats and two biological repeats.

### 2.5. Gene Expression

The cells were seeded as described in Section 2.4 and treated with the polysaccharide extracts and the corresponding level of solvent at the calculated IC_50_ dosage. RNA was isolated from the cells using an RNeasy Plus Mini Kit (Qiagen^®^, Bio-strategy Ltd., Auckland, New Zealand) after four doubling cycles. A Quantitect Reverse Transcription Kit (Qiagen^®^, Bio-strategy Ltd., Auckland, New Zealand) was used for the conversion of RNA samples to cDNA, which was diluted four times for RT-qPCR. RT-qPCR assays were performed according to the manufacturer’s manual (Ref TaqMan^®^ FA Mix manual). cDNA was amplified in a PCR process with the presence of a TaqMan^®^ Fast Advanced Master Mix (Applied Biosystems, Foster City, CA, USA) and TaqMan^®^ primer-sets (Applied Biosystems, Auckland, New Zealand). Details of the eleven TaqMan^®^ primer-sets used in this study are shown in Table 2. Glyceraldehyde 3-phosphate dehydrogenase (GAPDH) and hypoxanthine phosphoribosyltransferase (HPRT1) were selected as the two housekeeping genes for normalization purposes as their cycle threshold (CT) values were similar to those of the genes of interest. The meaningful differential gene expression was set at a cut off value of 2x [35]. PCR results were analysed using the 2^−ΔΔCT^ method [36].

### 2.6. AChE Inhibition

The AChE inhibition assay was performed based on Ellman and Benabent’s method [37,38] with slight modifications. Briefly, the sample (or control), AChE, Buffer A, and DTNB were added to the micro-wells in sequence at volumes of 30 μL, 30 μL, 30 μL, and 50 μL, respectively. The plates were placed in an incubator for 10 min at 37 °C and the initial reading was obtained by spectrophotometric measurement (EnSpire Multimode Reader (PerkinElmer, MA, USA)). Thereafter, each micro-well received 30 μL of ATCI and plates were placed in an incubator for 20 min at 37 °C to initiate the reaction. The reaction was stopped by pipetting 30 μL of SDS into the micro-wells. The absorbance was then measured and this measurement was regarded as the second reading.

A = second reading − initial reading(1)

AChE inhibition (%) = ((A (blank) − A (sample))/(A (blank))) × 100(2)

The inhibition of the samples at different concentrations was calculated by Equations (1) and (2). Eserine was used as a positive control. The samples (dissolved in water, ranging in concentration from 10.24–32.00 mg/mL for WCP and 3–16 mg/mL for BP1) along with eserine (ranging from 0.0625–1 μM) were analyzed at five different concentrations to calculate the IC_50_ value (concentration of test sample that inhibits 50% of AChE). The IC_50_ values were obtained from dose-effect curves generated through linear regression. BP2 was not tested as its solvents, either 0.2 N NaOH or DMSO, caused significant interference in this experiment.

### 2.7. Determination of Antioxidant Capacity

The antioxidant capacity of the polysaccharides relative to the antioxidant reference Trolox ((S)-(2)-6-hydroxy-2, 5, 7, 8-tetramethyl-chroman-2-carboxylic acid) was measured. WCP was dissolved in water; BP1 and BP2 were dissolved in 0.2 N NaOH. A calibration curve was prepared for each test below using Trolox as a standard. The absorbance was measured at the required wavelength by EnSpire Multimode Reader (PerkinElmer, MA, USA). The result of each experiment was expressed as micromole Trolox equivalent per gram of sample (µmol TE/g polysaccharides).

#### 2.7.1. DPPH Assay

DPPH scavenging activity of the polysaccharides was assessed in accordance with the method applied by Tang’s group [39] with minor modifications. Briefly, 10 µL of sample, standard, or water/0.2 N NaOH (blank) was added to 200 µL of DPPH solution. The absorbance of the solution was measured at 517 nm after 60 min of incubation in the dark and the decrease in the absorbance, triggered by the proton donating activity of the sample, standard, or solvent (blank), was calculated.

#### 2.7.2. FRAP

FRAP (Ferric Reducing Ability of Plasma) assaying was performed by a method modified from Benzie’s report [40]. An amount of 10 µL sample, standard, or water/0.2 N NaOH (blank) was added to 200 µL of the FRAP working solution. The absorbance of the solution was measured at 593 nm, after incubation in the dark, at room temperature for 60 min.

#### 2.7.3. ABTS

ABTS assaying was conducted in accordance with Du’s description [41]. Briefly, 10 µL of sample, standard, or water/0.2 N NaOH blank was added to 200 µL of radical reagent (ABTS working solution). The absorbance of the solution was measured at 734 nm, after incubation in the dark, at room temperature for 60 min. The decolorization caused by radical scavenging activity was then calculated.

### 2.8. Statistical Analysis

All experiments were carried out in at least three technical repeats and two biological repeats and the results were expressed as the mean of replicates ± pooled standard deviation. A one-way ANOVA was performed with IBM SPSS Statistics and differences between means at the *p* < 0.05 level were considered significant.

## 3. Results and Discussion

### 3.1. Polysaccharide Yield and Protein Content in Polysaccharides

The yields of WCP, BP1, and BP2 were 6.23, 4.21, and 4.31%, respectively. The sum of BP1 and BP2 was higher than WCP, indicating that most polysaccharides in H. novae-zealandiae were poorly soluble in water. The protein content of the three polysaccharide fractions were identified as 9.91, 13.35, and 2.27% in WCP, BP1 and BP2, respectively, indicating that a fraction of the extracted polysaccharides might be present as glycoprotein.

### 3.2. Proliferative Inhibition of PCa and HEK293 Cells

The calculated IC_50_ values of the polysaccharide fractions in the three PCa cell lines and the non-cancer control HEK293 cell line have been tabulated in Table 3. The data for BP2 are not listed due to the reason described in Section 2.4.

The lowest IC_50_ values for both WCP and BP1, in the four cell lines tested, were obtained in LNCaP. Specifically, the IC_50_ values of WCP were 0.96 and 0.61 mg/mL for PC3 and LNCaP cells, respectively. However, Table 3 shows that both polysaccharide fractions are anti-proliferative to the HEK293 cells. Although the anti-proliferative effect was weaker than that observed in PCa cells, this is not ideal. Nevertheless, it should be noted that the HEK293 cell line is not a non-cancer prostate cell line. BP1 was omitted in the gene expression experiment as it exhibited a weaker anti-proliferative activity WCP [42].

**Figure 1:**
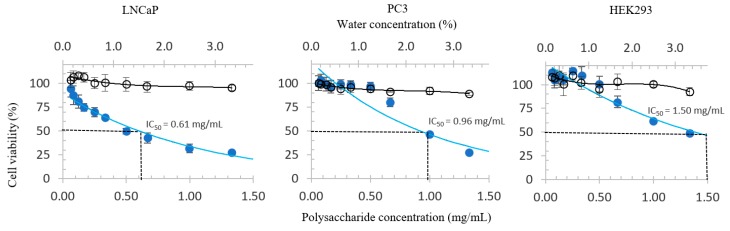
Dose-response curves of WCP in LNCaP, PC3, and HEK293 cell lines. The blue and black circles represent the growth inhibitory properties of the WCP and solvent control (water), respectively.

Figure 1 shows the dose-response curves of the three cell lines treated with WCP. The black lines in the figures represent the treatment control. Thanks to the relatively horizontal line observed, it can be inferred that the inhibitory activities of WCP may not attributed to its solvent.

### 3.3. RT-qPCR

The results of the RT-qPCR assays are presented in Table 4. Our screening shows that the polysaccharides are likely to affect the LNCaP and PC3 cells through different pathways. Specifically, we observed upregulation in the expression of *CASP3*, *CASP8*, and *CASP9* in the treated LNCaP cells by 2.10, 3.89, and 3.14 times relative to HPRT1, respectively. Meanwhile, similar increments were not observed in the treated PC3 cells. However, the expression of *IL6* in *PC3* was downregulated by 0.52 times relative to *HPRT1*, while the expression of *IL24* in the same cells was upregulated by 3.61 times relative to *HPRT1*.

#### 3.3.1. LNCaP

**Figure 2:**
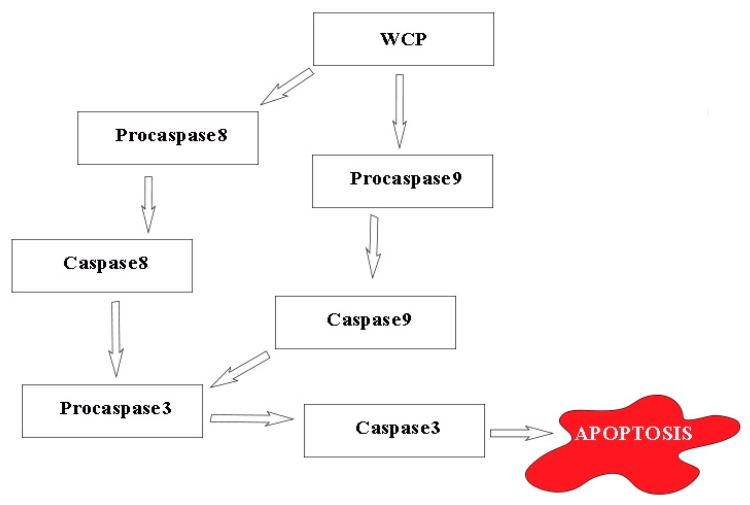
Proposed mechanism of the WCP-triggered, caspase-mediated apoptotic pathway in LNCaP cells, based on the results of the present study. Arrows indicate promoting.

The upregulation of *CASP3*, *CASP8*, and *CASP9* in LNCaP cells treated with WCP suggests the caspase-mediated apoptotic pathway. We theorize (Figure 2) that the WCP might upregulate the expression of *CASP8* and *CASP9* by cleaving their respective procaspases. Both CASP8 and CASP9 will then cleave procaspase3 into CASP3, which is thought to be an initiator of programmed cell death [43]. The activation of CASP3 is thought to initiate apoptosis through both the inactivation of essential cellular proteins (such as poly-adenosine diphosphate-ribose polymerase (PARP)) and also activation of nucleases that trigger DNA fragmentation [44].

#### 3.3.2. PC3

Unlike in LNCaP cells, PC3 cells treated with WCP did not exhibit meaningful changes in the expression of *CASP3*, *CASP8*, and *CASP9*. Nonetheless, meaningful changes in gene expression were found in the interleukin (IL)-encoding genes *IL6* and *IL24.* While *IL6* was downregulated by 0.52 times relative to *HPRT1*, *IL24* was upregulated by 3.61 times relative to *HPRT1* (Table 4). Since IL6 is pro-inflammatory and IL24 is anti-inflammatory, these results imply that the WCP polysaccharide fraction may exert an anti-inflammatory effect. Nonetheless, the pathway underlying this anti-inflammatory activity needs further investigation. Ultimately, it is worth mentioning that inflammation does not affect cancer in a direct manner but that it governs various pathways related to the hallmarks of cancer [45].

### 3.4. AChE Inhibition

WCP and BP1 were found to inhibit AChE in a concentration-dependent manner. This assay was not performed on BP2 as it was insoluble in the buffer solution. The IC_50_ of the positive control (eserine) was determined as 0.073 µM, which is comparable to the values reported by several authors, including 0.04 µM [42] and 0.025 µg/mL (equivalent to 0.091 µM) [46].

This assay revealed that both WCP and BP1 exerted weak AChE inhibitory activities, with IC_50_ values of approximately 8 mg/mL for both fractions as shown in Table 5. Conclusively, polysaccharides isolated from *H. novae-zealandiae* are not associated with memory improvement through the AChE inhibitory pathway. This result seems disappointing. However, the inhibition of AChE is not the only pathway through which to increase the content of Ach. A related study [47] has explored the neuroprotective effects of polysaccharide-enriched aqueous extract isolated from *H. erinaceus*. The results revealed that the extract improved the central cholinergic system function in Alzheimer’s mice by enhancing the acetylcholine and choline acetyltransferase concentrations in a dose-dependent way in both the serum and the hypothalamus. In addition, alternative mechanisms, such as the amyloidbeta hypothesis, should be further studied to investigate the potential amnesia prevention activity of polysaccharides isolated from *H. novae-zealandiae.*

### 3.5. Antioxidant Activities

It was observed that the polysaccharide fractions exhibited antioxidant activity in a concentration-dependent manner in all three antioxidant assays. The solvent of 0.2 N NaOH used for BP1 and BP2 was found to interfere with the readings of the DPPH assay, hence the DPPH data for BP1 and BP2 have not been included. WCP generated the highest result in the FRAP assay with a value of 48.24 µmol TE/g, while BP1 generated the highest result in the ABTS experiment with 83.95 µmol TE/g (Table 5).

DPPH, FRAP, and ABTS are three of the most frequently used methods to evaluate antioxidant activity of the candidate. As each assay has its specific mechanisms and limits, they are usually carried out together to achieve more reliable results. The results obtained from individual DPPH, FRAP, and ABTS experiments were relatively consistent, which makes it reasonable to assume that the polysaccharide fractions of *H. novae-zealandiae* have antioxidant potential. Nevertheless, it is worth noting that all the three assays are based on in vitro chemical reactions and not biological systems. The results, therefore, should be interpreted in context to avoid exaggeration. It is suggested that biological antioxidant assessment be performed as a further step to validate the antioxidant activity of the polysaccharide fractions of *H. novae-zealandiae.* This will provide an approach to the utilization of *H. novae-zealandiae* for oxidative-related diseases and justification for performing such tests in vivo.

## 4. Conclusions

This is, to the best of our knowledge, the first report on the potential health benefits of polysaccharides isolated from the New Zealand indigenous edible *H. novae-zealandiae.* Three polysaccharide fractions were examined for their anti-proliferative, AChE inhibitory, and antioxidant activities. Both the water-soluble fraction and one of the alkali soluble fractions exhibited meaningful anti-proliferative activity in LNCaP and PC3 cell lines, with the lowest IC_50_ value of 0.61 mg/mL obtained in LNCaP cells that were treated with the water-soluble polysaccharide fraction. The gene expression of *CASP3*, *CASP8*, and *CASP9* in treated LNCaP cells indicates that apoptosis may underlie the anti-proliferative activity of the WCP fraction. The other probable pathway, found in PC3, was anti-inflammation, supported by changes in the gene expression of *IL6* and *IL24* following treatment. This anti-inflammatory activity might not directly trigger the anti-proliferative effects; instead, it may suppress the signaling pathways of various cancer hallmarks. The polysaccharides of *H. novae-zealandiae* also exhibited AChE inhibition (though weak) and antioxidant activities. However, more studies revealing the possible mechanism of anti-PCa activity of polysaccharides from *H. novae-zealandiae* need to be performed to justify the application of *H. novae-zealandiae* in prostate cancer therapy. In addition, chemical analysis including molecular weight and carbohydrate composition of the polysaccharides should be carried out to allow a better understanding of the bioavailability and metabolic fate of these polysaccharides.

## Figures and Tables

**Table 1:** Dosing regimen of anti-proliferation assay. Legend: WCP, water-soluble polysaccharides; BP1, base-soluble polysaccharides.

Polysaccharides	1	2	3	4	5	6	7	8	9	10
WCP (mg/mL)	1.333	1.000	0.667	0.500	0.333	0.250	0.167	0.125	0.083	0.062
BP1 (mg/mL)	1.000	0.750	0.500	0.375	0.250	0.188	0.125	0.094	0.063	0.047
Water control (%)	3.333	2.500	1.666	1.250	0.833	0.625	0.417	0.312	0.228	0.156

**Table 2:** List of selected TaqMan^®^ primer-sets (Applied Biosystems) utilized in this study and their respective RT-PCR assay IDs with target chromosome locations.

Gene	Assay ID	Human Target Chromosome Location	Amplicon Length (bp)
*CASP3*	Hs00991555_m1	Chr.4: 184627696 - 184649475	118
*CASP8*	Hs01018160_m1	Chr.2: 201233443 - 201287711	124
*CASP9*	Hs00962278_m1	Chr.1: 15491401 - 15524912	84
*GAPDH*	Hs99999905_m1	Chr.12: 6534405 - 6538375	122
*HPRT1*	Hs99999909_m1	Chr.X: 134460145 - 134500668	100
*IL24*	Hs01114274_m1	Chr.1: 206897404 - 206904139	67
*IL6*	Hs00174131_m1	Chr.7: 22725889 - 22732002	95

**Table 3:** 50% growth inhibition (IC_50_) of prostate cancer (PCa) cells and HEK293 cell treated with WCP isolated from *H. novae-zealandiae.*

Polysaccharides	DU145	PC3	LNCaP	HEK293
WCP (mg/mL)	>> 1.33	0.96 ± 0.10	**0.61 ± 0.05** *	1.50 ± 0.12
BP1 (mg/mL)	>> 1.00	>> 1.00	**1.02 ± 0.12** *	1.12 ± 0.11
BP2 (mg/mL)	x	x	x	x

Values are reported as mean ± pooled SD. >> indicates that IC_50_ was not achieved, although anti-proliferative activity was observed relative to the water control; bold indicates the most powerful anti-proliferative activity among the four cell lines; x indicates that the experiment was not successful due to solvent interference of the polysaccharide fraction; * indicates significant difference at *p* < 0.05. Legend: BP2, base-polysaccharides.

**Table 4:** Normalized differential gene expression in cell lines after treatment with WCP from *H. novae-zealandiae*.

-	LNCaP		PC3	
	*GAPDH*	*HPRT1*	*GAPDH*	*HPRT1*
*CASP3*	1.18 ± 0.14	**2.10±0.15** *	1.44 ± 0.11	1.10 ± 0.15
*CASP8*	0.93 ± 0.79	**3.89±0.41** *	1.54 ± 0.11	1.49 ± 0.12
*CASP9*	1.45 ± 0.16	**3.14±0.26** *	0.79 ± 0.08	0.82 ± 0.09
*IL6*	X	X	1.02 ± 0.11	**0.52±0.05** *
*IL24*	X	X	**5.67±0.48** *	**3.61±0.29** *

The results have been normalized to water (solvent control) and are expressed as mean ± pooled SD; values in bold indicate exceeding the differential expression threshold of 2x up-regulation/down-regulation; x indicates that the gene was not amplified; * indicates significant difference at *p* < 0.05.

**Table 5:** Acetylcholinesterase (AChE) inhibitory and radical scavenging activities of polysaccharides isolated from *H. novae-zealandiae.*

Activity	WCP	BP1	BP2
AChE inhibition (mg/mL IC_50_)	8.26 ± 0.52	8.64 ± 0.45	x
DPPH (µmol TE/g)	59.74 ± 1.52	x	x
FRAP (µmol TE/g)	**48.24±1.27** *	41.49 ± 1.24	32.18 ± 0.97
ABTS (µmol TE/g)	65.58 ± 1.87	**83.95±2.59** *	58.35 ± 1.57

Values are reported as mean ± pooled SD. Antioxidant activity was assessed as micromole Trolox equivalents per gram of polysaccharide fraction (µmol TE/g). x indicates that the data were not obtained; bold indicates the highest antioxidant potential among values obtained from the three experiments; * indicates significant difference at *p* < 0.05. Legend: DPPH, 1,1-diphenyl-2-picrylhydrazyl; ABTS, 2,2’-azino-bis(3-ethylbenzothiazoline-6-sulphonic acid).
